# The Impact of Artificial Intelligence on Optimizing Diagnosis and Treatment Plans for Rare Genetic Disorders

**DOI:** 10.7759/cureus.46860

**Published:** 2023-10-11

**Authors:** Shenouda Abdallah, Mouhammad Sharifa, Mohammed Khaleel I.KH. Almadhoun, Muhammad Muneeb Khawar, Unzla Shaikh, Khaled M Balabel, Inam Saleh, Amima Manzoor, Arun Kumar Mandal, Osatohanmwen Ekomwereren, Wai Mon Khine, Oluwaseyi T. Oyelaja

**Affiliations:** 1 Surgery, Jaber Al Ahmad Al Jaber Al Sabah Hospital, Kuwait City, KWT; 2 Medicine, University of Aleppo, Aleppo, SYR; 3 Medicine and Surgery, Mutah University, Karak, JOR; 4 Internal Medicine, Mayo Hospital, Lahore, PAK; 5 Internal Medicine, Liaquat University of Medical and Health Sciences, Hyderabad, PAK; 6 Internal Medicine, Saint Joseph Regional Center, Mishwaka, USA; 7 Pediatrics, University of Kentucky College of Medicine, Lexington, USA; 8 Internal Medicine, Jinnah Sindh Medical University, Karachi, PAK; 9 General Medicine, Mahawai Basic Hospital/The Oda Foundation, Kalikot, NPL; 10 Medicine, Manipal College of Medical Sciences, Pokhara, NPL; 11 Trauma and Orthopaedics, Royal Shrewsbury Hospital, Shrewsbury and Telford Hospital NHS Trust, Shrewsbury, GBR; 12 Internal Medicine, Caribbean Medical School, St. Georges, GRD; 13 Surgery, Wyckoff Heights Hospital, Brooklyn, USA

**Keywords:** genetic basis of congenital heart disease, genetic variant, rare genetic diseases, deep learning artificial intelligence, genetic syndromes

## Abstract

Rare genetic disorders (RDs), characterized by their low prevalence and diagnostic complexities, present significant challenges to healthcare systems. This article explores the transformative impact of artificial intelligence (AI) and machine learning (ML) in addressing these challenges. It emphasizes the need for accurate and early diagnosis of RDs, often hindered by genetic and clinical heterogeneity. This article discusses how AI and ML are reshaping healthcare, providing examples of their effectiveness in disease diagnosis, prognosis, image analysis, and drug repurposing. It highlights AI's ability to efficiently analyze extensive datasets and expedite diagnosis, showcasing case studies like Face2Gene. Furthermore, the article explores how AI tailors treatment plans for RDs, leveraging ML and deep learning (DL) to create personalized therapeutic regimens. It emphasizes AI's role in drug discovery, including the identification of potential candidates for rare disease treatments. Challenges and limitations related to AI in healthcare, including ethical, legal, technical, and human aspects, are addressed. This article underscores the importance of data ethics, privacy, and algorithmic fairness, as well as the need for standardized evaluation techniques and transparency in AI research. It highlights second-generation AI systems that prioritize patient-centric care, efficient patient recruitment for clinical trials, and the significance of high-quality data. The integration of AI with telemedicine, the growth of health databases, and the potential for personalized therapeutic recommendations are identified as promising directions for the field. In summary, this article provides a comprehensive exploration of how AI and ML are revolutionizing the diagnosis and treatment of RDs, addressing challenges while considering ethical implications in this rapidly evolving healthcare landscape.

## Introduction and background

Rare genetic disorders (RDs), characterized by their low prevalence (typically affecting fewer than 1 in 2000 individuals in the European Union) and encompassing approximately 8000 identified conditions worldwide, impose a significant burden on both individuals and healthcare systems. Some rare diseases manifest in childhood (e.g., infantile spinal muscular atrophy), while others remain dormant until adulthood (e.g., amyotrophic lateral sclerosis and thyroid cancer), making diagnosis and treatment complex. Limited understanding of their pathophysiological mechanisms and the challenges of developing therapies for small patient groups contribute to the lack of effective treatments for most rare diseases [1].

Affecting an estimated 8 to 10% of the global population, RDs demand increased attention and dedicated resources [2]. However, the field faces significant challenges, including underestimation of disease prevalence, limited funding, dispersed patient populations, limited insights into disease mechanisms, and limited interest from pharmaceutical companies [3]. The path to proper diagnosis and treatment for individuals with RDs is riddled with challenges. Clinicians often lack the specialized expertise needed to recognize and manage these conditions, leading to suboptimal treatment or misdiagnosis. Overlapping clinical features and insufficient molecular data further complicate the diagnostic process, emphasizing the importance of early and accurate diagnoses [2]. Treatment options are often limited to symptomatic or repurposed interventions, leaving many patients without approved therapeutic options [4,5].

Despite these obstacles, recent advancements in genetic research provide hope for those affected by these disorders. Advanced sequencing techniques and global databases have enabled the identification of causative genetic mutations, offering prospects for precise diagnosis and personalized treatment. Genomic analysis technologies, such as next-generation sequencing and "omics" technologies, have improved our ability to diagnose and understand rare diseases [3]. Data obtained from these technologies signify a significant surge in data volume, necessitating the processes of selection, analysis, and integration [6]. This era of "big data" presents a substantial opportunity for advancing research and development in the field of therapy for RDs [3].

Artificial intelligence (AI), particularly machine learning (ML), emerges as a powerful tool in addressing these challenges. AI has already demonstrated its effectiveness in various biomedical and clinical contexts, including the COVID-19 pandemic [7]. ML and deep learning (DL) models have been instrumental in early COVID-19 detection by analyzing patient demographic, clinical, and epidemiological data. Furthermore, they have been essential in the development of diagnostic tools that swiftly analyze CT scans and X-rays to identify patterns indicative of the disease [8]. AI's predictive capabilities extend to assessing patient vulnerability for tailored treatment and expediting vaccine discovery. Moreover, AI aids public health initiatives through contact tracing, monitoring virus spread, and predictive modeling for outbreak identification. In rare disease management, AI contributes to precision medicine by utilizing extensive data from patient registries to uncover potential associations, thereby enhancing prevention, diagnosis, treatment, and monitoring based on individual genetic profiles [9].

This narrative review article aims to comprehensively explore the role of AI and ML in improving the diagnosis and treatment of RDs. It covers the complexities of RDs, the importance of accurate diagnosis and tailored therapies, the historical evolution and recent advancements in AI/ML in healthcare, and their potential benefits and challenges. The review discusses how AI/ML uses genetic and clinical data for precise diagnosis, includes case studies of successful applications, and examines personalized medicine and drug discovery for RDs. Ethical considerations related to AI-driven treatment decisions are addressed, along with challenges such as data quality, algorithm interpretability, regulatory concerns, and AI/ML integration into healthcare systems. The review also explores future directions, emerging trends, and potential impacts, such as AI-powered telemedicine and collaboration between researchers, clinicians, and AI experts, to highlight the transformative potential of AI/ML/DL in RDs. This narrative review has been synthesized by reviewing the relevant articles from PubMed and Google Scholar.

## Review

RDs: a complex challenge

Despite being a prominent topic in global health discussions, there is no universally accepted definition for rare diseases, with health systems often relying on criteria related to prevalence or the number of affected individuals. Rare genetic diseases, affecting around 8% of the global population, encompass a diverse array of conditions, with an estimated 6000 to 8000 distinct diseases. The lack of specific therapies for 95% of these conditions amplifies their impact, categorizing them as "orphan diseases." Approximately 80% of rare diseases have a genetic origin and persist throughout an individual's life, even if symptoms are not immediately evident [10].

The intricacies intrinsic to rare genetic diseases extend beyond their genetic underpinnings, encompassing degenerative and chronically debilitating attributes that cast a wide-ranging impact not only on physical health but also on the mental, sensory, and behavioral capacities of afflicted individuals and their families [11]. This complexity underscores the imperative for comprehensive and sustained care tailored to the unique characteristics of each disease and its repercussions on the lives of affected individuals [10].

The rarity associated with these individual genetic diseases represents only one facet of their complexity. Much of the intricacy arises from the amalgamation of genetic and clinical heterogeneity [12]. This implies that analogous clinical presentations may stem from divergent genetic mechanisms, encompassing locus or allele heterogeneity, where mutations may occur in distinct genes or involve diverse alleles within the same gene [13,14]. Clinical heterogeneity further confounds the landscape, as identical genetic mutations may manifest in various disease phenotypes. Furthermore, the penetrance of genetic predisposition fluctuates, and phenotypic expression exhibits a high degree of variability. The lack of specificity in clinical symptoms for genetic diseases further compounds the challenge of accurate identification of these rare conditions [12].

The rarity of genetic diseases presents an additional challenge for establishing diagnoses, particularly in countries with smaller populations where healthcare workers, even at tertiary health institutions, may lack familiarity with rare diseases, resulting in deficits in infrastructure, coordination, resources, and knowledge. As a result, the diagnosis of rare genetic diseases was historically somewhat inefficient [15]. The previous gold standard approach of Sanger sequencing could only analyze single genes. In cases of genetic heterogeneity, a time-consuming and expensive "gene-by-gene" testing approach was employed, often unavailable in some health systems. Furthermore, accurate diagnostic hypotheses were crucial for successful genetic diagnosis [16-18].

The journey of individuals afflicted with rare genetic diseases is marked by protracted and arduous diagnostic processes. In a quarter of cases, patients endure between 5 and 30 years after the onset of their illness before obtaining a correct diagnosis, necessitating the involvement of a proficient and comprehensive clinical team [19]. A survey conducted by the National Organization for Rare Disorders revealed that 50% of patients and caregivers attribute diagnostic delays to a lack of understanding about the disease, while 42% believe that delays stem from a shortage of medical specialization [9]. Many patients identify issues with doctors' ability to connect symptoms, especially across different organ systems, in addition to extended waiting times to see specialists and the need for additional tests. The delay in diagnosis can profoundly impact a patient's clinical condition, making prompt and accurate diagnosis the crucial starting point for accessing therapeutic interventions and resources that can ensure a positive clinical outcome [20].

Nevertheless, the complexity does not conclude with diagnosis; it extends to prognosis and treatment [21]. Challenges in prognostication are intrinsically linked to the absence of reliable parameters and biomarkers, as the molecular pathophysiological mechanisms remain largely uncharted. Furthermore, the limited patient pool hampers the derivation of statistically significant parameters [22]. As a result, patients' prognoses fluctuate based on diverse genetic and environmental factors, complicating the establishment of treatment and rehabilitation standards. The development of new drugs and treatments is also hindered by research costs, which are high, and revenues, which are meager due to the limited patient pool. The heterogeneous patient populations, unknown etiology and pathogenesis, variability in disease progression timing, and the absence of comprehensive clinical studies converge to make the quest for specific drugs exceedingly challenging [9].

The emergence of AI and ML in healthcare

AI and ML have emerged as transformative forces within the healthcare domain, extending well beyond conventional diagnostic and therapeutic methodologies. These technologies, although often used interchangeably, possess distinct attributes and are collectively reshaping medical decision-making through their capacity to harness extensive datasets and computational capabilities [23].

At the core of this transformation lies the concept of AI, a multifaceted domain encompassing a broad spectrum of systems and technologies capable of executing tasks that mimic human cognitive processes. ML, conversely, operates within the confines of AI, leveraging large datasets to acquire knowledge, enhance task efficiency, and make informed predictions. DL, a subset of ML, delves even deeper into the realm of artificial neural networks [24,25]. All three entities, AI, ML, and DL, demand substantial computational resources and specialized training, surpassing human capabilities in processing vast datasets efficiently [26].

Within the healthcare landscape, the integration of AI is already evident, with applications ranging from disease diagnosis to drug dosage calculations. The benefits are myriad, encompassing enhanced efficiency, precision, cost-effectiveness, and reduced workload for medical professionals. Impressively, AI has demonstrated its ability to match or even surpass human accuracy in medical decision-making across various domains [27]. For instance, deep neural networks have rivaled the diagnostic proficiency of dermatologists in the analysis of biopsy images, showcasing their potential to augment the expertise of medical practitioners [28]. By harnessing extensive datasets from sources like UNOS and genetic registries, ML can predict short- and long-term outcomes with superior accuracy compared to current practices. Furthermore, as implantable and wearable medical technology continues to advance, ML can facilitate real-time patient monitoring, potentially reducing hospitalizations and enhancing post-operative care. This convergence of technology and healthcare holds the promise of not only improving patient outcomes but also alleviating the burden on healthcare systems by streamlining processes and enhancing overall efficiency [23].

The influence of AI and ML in healthcare extends across various facets of the industry. These technologies encompass a wide array of applications, including precision medicine, predictive modeling, and image analysis. ML, with its neural networks and statistical models, plays a pivotal role in categorizing health information and expediting decision-making. AI also significantly contributes to disease diagnosis, utilizing extensive datasets, patient histories, and medical imaging to assist healthcare professionals in making accurate and timely decisions [29]. Whether detecting cancerous lesions in medical images or predicting disease onset based on patient attributes, AI has proven its mettle in enhancing early disease diagnosis, a critical factor in saving lives [30,31]. Furthermore, AI plays a pivotal role in disease prognosis by estimating disease progression, survival rates, and risk assessments. Neural network models and AI algorithms are deployed to predict survival rates in various diseases, further empowering medical practitioners with valuable insights into patient outcomes. Beyond diagnosis and prognosis, AI excels in disease characterization, identifying influential predictors in specific conditions, thereby contributing to our understanding of diseases such as narcolepsy [32]. Drug repurposing also benefits significantly from AI, which recognizes patterns in drug targets and proposes potential candidates for the treatment of rare genetic diseases, thereby expanding therapeutic options [33].

Another dimension of AI's influence in healthcare is its ability to establish disease associations and clustering through networks built on phenotypic features. These networks reveal unexpected relationships between diseases, enhancing our comprehension of genetic disorders and potentially leading to breakthroughs in treatment and management [32]. In the context of clinical trials, where patient identification and recruitment can be challenging, AI-based methods excel by facilitating the creation of virtual clinical trials. This innovative approach streamlines the testing of treatments for rare genetic diseases and other conditions, ultimately accelerating the pace of medical research and improving access to potentially life-saving interventions [34]. Based on numerous real-world examples of AI applications, it is evident that AI possesses a vast and diverse spectrum of potential uses. These applications span from the most straightforward enhancements in operational processes to the most intricate and advanced emergency patient therapies [35].

Leveraging AI/ML for the diagnosis of RDs

Advancements in the realm of AI and ML hold considerable potential for enhancing the diagnosis and management of rare diseases. AI and ML rely upon extensive datasets to train algorithms, enabling them to make predictions, such as the classification of tumors in medical imagery. These technologies are increasingly integrated into the field of healthcare and, in many instances, exhibit capabilities that are on par with or surpass human-level performance [36].

Rare diseases, characterized by their unique diagnostic and therapeutic complexities, stand to derive substantial benefits from the application of AI and ML. It is infeasible for healthcare practitioners to memorize information pertaining to the multitude of rare diseases, yet computers are adept at storing and analyzing vast quantities of digital data. AI systems can effectively harness this data, facilitating tasks like patient categorization into disease groups or prognostic predictions. For example, an AI expert system capable of calculating disease probabilities based on patient symptoms can expedite the diagnosis of rare diseases [37]. Another exemplar, Face2Gene, employs computer vision and DL techniques to aid in the diagnosis of rare genetic conditions by analyzing facial photographs of patients [38].

AI has made significant inroads into the healthcare sector, playing a pivotal role in tasks encompassing image analysis, surgery scheduling, diagnosis in resource-constrained regions, and the efficient management of extensive patient datasets [39]. Within the field of oncology, AI has exhibited its capabilities by proficiently analyzing tumor images and decoding DNA sequences to identify genetic abnormalities. Notably, research has demonstrated that DL systems can rival dermatologists in accurately identifying skin lesions [40]. This breakthrough offers significant promise for early cancer detection, extending diagnostic capabilities beyond the confines of traditional clinical settings. AI-driven devices, including smartphones, have the potential to detect tumors, democratizing access to dermatologist-level diagnosis [41]. Moreover, DL extends its capabilities to encompass the identification of cancer within whole-slide images and the delineation of intricate molecular characteristics of tumors, including the expression levels of marker proteins [40]. These capabilities empower healthcare professionals to gain profound insights into the biological profiles of tumors, ultimately advancing the early detection and diagnosis of cancer [41].

AI's utility transcends visual analysis, proving to be a formidable asset in the identification of genetic mutations linked to hereditary diseases. Leveraging next-generation sequencing (NGS), a technique capable of sifting through millions of DNA sequences and identifying thousands of genetic anomalies within a single tumor sample, AI possesses the capacity to process this voluminous data [39]. By efficiently discerning the significance of various mutation combinations in individuals afflicted by genetic diseases, AI markedly enhances the accuracy and speed of disease detection and diagnosis [42]. These tasks would be herculean for human operators, given the sheer magnitude of the data involved.

Furthermore, AI emerges as a game-changing force in the diagnosis of rare genetic diseases. Statistics indicate that approximately 30 million Americans are afflicted by rare diseases, with a staggering 40% experiencing misdiagnoses [22]. Often, the delayed diagnosis of RDs is attributed to a dearth of information and awareness surrounding these conditions [43]. In this context, AI emerges as a potential solution by adeptly deciphering extensive datasets from diverse sources. This analytical acumen empowers AI to make significant contributions to the accurate diagnosis of individuals grappling with rare diseases, thereby mitigating the challenges posed by limited information and awareness associated with conventional diagnostic approaches [41].

Tailoring treatment plans with AI/ML

The pursuit of innovative treatments for rare diseases has long posed a pressing and formidable challenge, primarily due to the scarcity of essential data encompassing drug molecules, genetic information, and protein structures. This challenge is further compounded by the rapid pace of the generation of biomedical knowledge, rendering the linkage between disease mechanisms and potential therapeutic interventions increasingly intricate [9]. It is striking to note that nearly 95% of rare diseases currently lack the approval of the Food and Drug Administration (FDA) for specific pharmaceutical treatments. As the number of rare disease diagnoses continues to surge, the urgency to characterize these conditions and align patients with appropriate therapeutic modalities escalates [44].

In the face of these formidable challenges, the realm of AI offers a glimmer of hope by furnishing a means to transform vast volumes of biomedical knowledge into actionable insights for the identification of therapeutic strategies [9]. The Hugh Kaul Precision Medicine Institute has introduced an innovative AI platform named mediKanren, which is underpinned by knowledge graphs. This pioneering tool harnesses mechanistic insights into genetic disorders to streamline the integration of pertinent literature and databases, presenting a scalable approach that has already rendered benefits to over 500 families affected by rare diseases [45]. It leverages AI's capabilities to decipher complex biomedical knowledge and present it in an easily searchable format, thereby assisting healthcare professionals and researchers in comprehending rare diseases and pinpointing potential therapeutic avenues [9]. 

Within the sphere of RD treatment, ML and DL have arisen as indispensable tools. DL facilitates the creation of more customized therapeutic regimens, while ML finds its utility predominantly in the realm of clinical trials. DL algorithms, such as Support Vector Machine and Random Forest, excel in analyzing complex, high-dimensional data and are commonly employed to study rare medical conditions with limited datasets. They enhance our understanding of these conditions, aiding in the identification of precise treatment targets. Furthermore, AI leverages large datasets from quantitative structure-activity relationship (QSAR) modeling and high-throughput screening to advance therapeutic development, including the design of novel compounds with improved properties [46]. High-throughput screening campaigns yield an extensive corpus of data, and this wealth of information has notably culminated in the discovery of pharmaceuticals like riluzole, effectively employed in the treatment of amyotrophic lateral sclerosis [47].

Challenges and limitations

The integration of AI into the healthcare domain presents a myriad of challenges that encompass ethical, legal, technical, and human dimensions [48]. Ethical quandaries emerge, primarily concerning data ethics, privacy, data ownership, and equity, thereby raising pertinent questions regarding the management of personal health data and potential biases inherent in AI systems [49,50]. Additionally, the incorporation of AI into healthcare precipitates ethical dilemmas for healthcare practitioners, as it may grapple with intricate clinical decisions and potentially magnify prejudiced findings, subsequently culminating in inaccurate risk assessments [51,52]. Legal complexities ensue from the dearth of comprehensive global AI regulations, rendering the establishment of clear responsibilities within AI healthcare applications a formidable undertaking. The preservation of security is paramount to preclude data breaches and vulnerabilities within AI systems, as malevolent attacks on these systems may lead to erroneous diagnostic outcomes. Furthermore, apprehensions arise regarding the safeguarding of human genetic resources and the preclusion of misappropriation of genetic information. Societal acceptance of AI in healthcare exhibits variance, with patients often exhibiting greater confidence in physicians compared to AI-generated diagnoses [48]. Moreover, healthcare personnel in less developed regions are apprehensive about the security of their employment as AI adoption escalates [53].

Scientific predicaments encompass challenges such as dataset shifts, algorithmic fairness, and adaptability to the ever-evolving healthcare milieu. AI models must demonstrate a capacity to accommodate dynamic alterations in patient demographics and clinical practices, while concurrently surmounting biases inherent in AI algorithms to facilitate the equitable delivery of healthcare services [54]. Additionally, concerns loom over data protection and ownership, as patients may exhibit reticence toward undergoing AI-based assessments, apprehensive about the potential misuse of their sensitive information. Thus, it is incumbent upon informed consent forms to explicitly elucidate the purposes of data utilization, thereby providing a robust legal shield [55]. The omission of rare diseases from AI training datasets may be detrimental, engendering biases and precluding equitable representation, thus contravening ethical principles [56]. Stigmatization must be forestalled to ensure that AI-generated health information does not detrimentally impact patients' prospects [57].

The issue of consent concerning the disclosure of incidental findings by AI systems warrants meticulous attention. AI may unveil unforeseen information during the course of medical imaging studies, engendering ethical quandaries regarding whether and to what extent such findings should be communicated to the concerned parties [58]. Ensuring transparent communication regarding the utilization of AI-generated findings assumes paramount significance in navigating these intricate ethical conundrums. In the specific context of rare diseases and AI, ethical considerations ascend to the forefront. Apprehensions of post-deployment misuse or misappropriation of information generated by AI systems underscore the exigency of stringent safeguards. AI must not wield personal health data to the detriment of patients, particularly those afflicted by rare diseases who already contend with heightened vulnerability. Effectively addressing these multifaceted challenges is indispensable to ensure the judicious and efficacious assimilation of AI into the realm of healthcare [55].

To ensure the fostering of trust and the adoption of AI within the medical community, the conduction of peer-reviewed randomized controlled trials (RCTs) is imperative. Nevertheless, it is noteworthy that certain RCTs have yielded divergent outcomes, underscoring the intricate interplay between AI and clinical practice. Attaining a high standard of reporting in AI research is indispensable for the meticulous assessment of potential biases and the utility of prediction models [54]. Adherence to established best practices, exemplified by the transparent reporting of multivariable prediction models (TRIPOD), is fundamental [59]. The metrics employed in the evaluation of AI systems necessitate alignment with clinical applicability, with the deployment of methodologies like decision curve analysis serving as a means to quantitatively ascertain the net benefit of AI models in informing clinical decisions [60]. Equally vital is the imperative to standardize evaluation techniques and test datasets across various studies to ensure equitable comparisons [54].

Future directions and potential impact

The prospective role of AI and ML in the domain of RDs holds substantial potential. Advanced technologies, including sentiment analysis and AI-ML tools, are positioned to augment brand visibility and customer engagement on social media platforms. When harmonized with AI-ML capabilities, social media transforms into a valuable instrument for data acquisition [61]. It is imperative, however, to underscore the significance of privacy safeguards through privacy-enhancing technologies (PETs) when harnessing this potential [62]. 

Second-generation AI systems are catalyzing a transformation in the treatment and management of RDs. Adhering to a patient-centric approach, these systems function as intermediaries that bridge diagnostic, prognostic, and therapeutic gaps. They deploy tailored closed-loop systems to enhance organ functionality, effectively addressing challenges associated with treatment tolerance and efficacy [9]. In light of the heterogeneity and complexity intrinsic to RDs, these AI-driven systems offer bespoke solutions that are indispensable. Notably, they play a pivotal role in the early identification and resolution of issues pertaining to treatment response. Significant strides in therapeutic and monitoring tools are pivotal to the care of individuals with RDs [46]. Second-generation AI tools demonstrate adaptability in the formulation of treatment regimens based on patient responses, electronic data, and patient-reported outcomes. They provide timely reminders for medication dosages, incorporate non-pharmacological therapeutic modalities, and optimize treatment timing based on response patterns [22]. For instance, given the variable response of patients with Gaucher disease to therapies, this monitoring framework serves as a crucial deterrent against the development of long-term complications [46].

Moreover, AI offers innovative solutions to circumvent challenges stemming from the rarity of these disorders. Traditional randomized clinical trials (RCTs) may often prove unviable owing to the infrequency of RD cases. AI-driven data mining and computable phenotype algorithms streamline the process of patient recruitment, significantly enhancing efficiency [3]. Prospective applications of AI as a synthetic control, leveraging data pertaining to disease progression, serve as an innovative approach poised to address challenges linked to patient enrollment. The identification of reliable biomarkers assumes paramount importance in the realm of therapy development. However, the attainment of regulatory approvals for these biomarkers remains ensnared in complexities [46].

The success of AI applications hinges upon the quality and quantity of available data. While methods like text mining hold promise, challenges related to data quality, the diverse nature of electronic medical records (EMR), and the presence of incomplete information pose obstacles to achieving semantic interoperability [55]. In contrast, medical imaging data, such as magnetic resonance (MR), computed tomography (CT), positron emission tomography (PET), and single photon emission CT (SPECT) scans, often adhere to standardized formats and exhibit greater consistency [36]. These images offer a wealth of information for the training of AI algorithms, particularly multivariate imaging data, such as total body PET (TB-PET), which enables visualization of multiple organ systems crucial for the diagnosis of rare diseases. This standardized and extensive imaging data holds substantial promise for the training of AI systems within the context of rare diseases [55].

The role of AI in direct patient care is currently undergoing evolution. The establishment of a universal standard is imperative, necessitating clear delineation of the boundaries within which AI operates, transparent communication with patients to obtain informed consent, and comprehensive evaluations of AI implementations. Emphasis on algorithmic transparency, the implementation of privacy safeguards, consideration of stakeholder interests, and the mitigation of cybersecurity risks all assume critical significance in the quest to minimize vulnerabilities [63].

AI has the potential to synergize with telemedicine, thereby facilitating virtual consultations and harnessing the capabilities of the Internet of Medical Things. The burgeoning expansion of health databases augments the resources accessible to AI research [63]. These repositories empower the development of sophisticated AI models capable of offering personalized therapeutic recommendations, precision dosage calculations, surveillance strategies, and the consideration of pivotal healthcare aspects [64].

## Conclusions

In conclusion, the integration of AI and ML into the realm of RDs represents a beacon of hope, offering transformative advancements in diagnosis, treatment, and patient care. RDs, which have long posed complex challenges due to their limited prevalence and diverse manifestations, are now on the cusp of a revolutionary transformation thanks to the convergence of AI and ML with vast biomedical knowledge and data. In the diagnostic realm, AI shines by swiftly processing extensive datasets from various sources, enabling the rapid identification of rare diseases. These technologies can categorize patients into disease groups, analyze symptoms, and even employ facial photographs for diagnosis, effectively mitigating challenges arising from limited information and awareness. Furthermore, AI empowers precision medicine by harnessing data from patient registries, facilitating personalized treatment, and monitoring based on individual genetic profiles. This not only enhances prevention, diagnosis, and treatment but also provides invaluable insights into patient outcomes.

In the pursuit of innovative treatments for RDs, AI-driven tools, including second-generation AI systems, play pivotal roles. These systems adopt a patient-centric approach, delivering tailored treatment regimens, optimizing medication dosages, and integrating non-pharmacological therapeutic modalities. They enhance treatment efficacy and minimize adverse effects, addressing the unique complexities of managing RDs. AI's influence extends beyond diagnosis and treatment to streamline clinical trials, making patient recruitment more efficient and feasible. Additionally, AI facilitates the identification of reliable biomarkers, paving the way for groundbreaking therapy development. Nonetheless, the integration of AI into healthcare presents challenges and limitations encompassing ethical, legal, and technical aspects, such as data ethics, privacy, bias, and security. Navigating these issues while ensuring transparent communication and addressing data protection concerns remains paramount.

Despite these challenges, the future of AI and ML in RDs holds immense promise. As AI applications continue to evolve, coupled with the expansion of health databases and the synergistic potential with telemedicine, personalized, efficient, and patient-centric care is becoming a reality. AI stands poised to significantly enhance the lives of individuals affected by RDs, ultimately alleviating the burden on both patients and healthcare systems.
